# Psychometric comparison of the Persian Salzburg Emotional Eating Scale and Emotional Eater Questionnaire among Iranian adults

**DOI:** 10.1186/s40337-022-00541-w

**Published:** 2022-02-05

**Authors:** Sahar Ghafouri, Abbas Abdollahi, Wanich Suksatan, Supat Chupradit, Aleiia J. N. Asmundson, Lakshmi Thangavelu

**Affiliations:** 1 Department of Counseling, Faculty of Education and Psychology, Alzahra University, Tehran, Iran; 2 Faculty of Nursing, HRH Princess Chulabhorn College of Medical Science, Chulabhorn Royal Academy, Bangkok, Thailand; 3 Department of Occupational Therapy, Faculty of Associated Medical Sciences, Chiang Mai University, Chiang Mai, 50200 Thailand; 4 Faculty of Psychology, University of Calgary, Alberta, Canada; 5 Center for Transdisciplinary Research, Department of Pharmacology, Saveetha Dental College, Saveetha Institute of Medical and Technical Science, Saveetha University, Chennai, India

**Keywords:** Salzburg Emotional Eating Scale, Emotional Eater Questionnaire, Psychometric assessment, Validation, Reliability, Adults

## Abstract

**Background:**

The Salzburg Emotional Eating Scale (SEES) and the Emotional Eater Questionnaire (EEQ) are self-reported measures developed to evaluate emotional eating in adults in Western countries. To date, the psychometric properties of the SEES and the EEQ have not been studied among Iranian adults. The aim of the current study is to translate the SEES and the EEQ from English to Persian and examine the psychometric properties of the SEES and EEQ.

**Method:**

The sample of this study comprised of 489 Iranian adults who completed the SEES and the EEQ questionnaires online.

**Results:**

Findings of face, content, and construct validity tests confirmed that the SEES and the EEQ had acceptable validity and appropriate reliability. The results from confirmatory factor analysis showed acceptable goodness-of-fit indices for two measures.

**Conclusion:**

Results of Average Variance Extracted, Construct Reliability, and goodness-of-fit indices showed that the SEES was better for evaluating emotional eating among Iranian adults than the EEQ.

## Plain English summary

Emotional eating is defined as overeating in response to negative emotions. Emotional eating could lead to substantial psychological suffering as well as health issues. Therefore, measuring emotional eating is important. This study aims to examine the psychometric properties of the Salzburg Emotional Eating Scale (SEES) and the Emotional Eater Questionnaire (EEQ) among Iranian adults. Participants in the study included 489 adults who completed both questionnaires online. The results showed that the SEES and the EEQ had acceptable face and content validity; however, the SEES showed better goodness-of-fit indices and factor loading values than the EEQ. Although the SEES and the EEQ are suitable for measuring emotional eating, the SEES showed better results and the SEES is recommended for measuring emotional eating among Iranian adults.

## Background

Food choices are likely to influence moods, and moods are likely to influence food choices. Where there is a constant relationship between food choices and mood, the effect of one is likely to have a reinforcing effect on the other. This could be due to a reduction in a bad mood (i.e., by satisfying hunger, or showing an improvement in one's mood as a result of sensory pleasure) [1]. It is believed that eating to modify unpleasant moods and emotions is a typical occurrence among many adults. Individuals, on the other hand, vary greatly in the amounts of food they consume to boost their mood, ranging from modest amounts of food on occasion to massive amounts during binge-eating episodes [2].

Emotional eating is a maladaptive coping mechanism for people who are experiencing negative emotions [3]. The word representation of emotional eating should be broadened to cover all possible pairings of emotions and intake styles, such that emotional eating may be described as a shift in consumer intake. Emotional eating such as this might include eating less or eating more than usual, in reaction to emotional statuses (which can include pleasure or unpleasant emotions) [4]. Emotional eaters may have fewer emotion-regulation strategies that effectively help them cope with their emotions [5].

Some factors that play a role in creating emotional eating are lower parental quality in infancy [6, 7], parental rejection [8], or childhood invalidation and emotional reactivity [9]. The side effects of emotional eating include overeating and binge eating, with the latter being linked to a more serious eating disorder [10, 11] or depression and weight gain [12].

Emotional eating, which develops as a psychological support for coping with negative emotions in children and young people, is on the rise [3]. Inadequate parenting and a high levels of depressed moods may be linked to increases in emotional eating throughout adolescence when combined with a genetic susceptibility [12]. In addition, during the coronavirus disease 2019 (COVID-19) pandemic, psychological distress symptoms and negative impact [13], aversion to ambiguity, fear of COVID-19, and depression [14] contributed to emotional eating.

There are various self-report measures that assess emotional eating [15], such as the Dutch Eating Behavior Questionnaire [16], the Emotional Eating Scale-II [17], the Emotional Overeating Questionnaire [18], the Emotional Appetite Questionnaire [19], the Three Factor Eating Questionnaire [20], and the Positive–Negative Emotional Eating Scale [10]. Among these, only the EMAQ addresses the difference between positive and negative feelings in emotional eating (although, the items were not developed based on experimental data and don't differ between types of emotions).

As a result, Meule, Reichenberger, and Blechert [21] developed the SEES. The SEES has 20 items which cover four subscales (*happiness, sadness*, *anger, and anxiety*). These subscales assess emotional eating by considering the differentiation between positive and negative emotions and high and low food consumption in emotional situations. In this study, a second questionnaire was used to conduct the psychometric study. The second questionnaire was developed by Garaulet and colleagues [22] and was named the EEQ. In this questionnaire, 10 items cover three subscales (*disinhibition, type of food, and guilt*). These subscales assess emotional eating in the Spanish population.

The previously mentioned questionnaires, such as the EES II, assessed eating disorders whereas the TFEQ had a larger number of items on the questionnaire itself, (i.e. 51 items).However, the EEQ assesses emotional eating, using 10 items as well as the SEES, using 20 items were selected for psychometric examination in this study.

An Iranian study showed that BMI and weight have increased over the last 20 years and the prevalence of overweight and obesity were 24.8% and 8%, respectively [23]. Another Iranian study showed that girls are more likely involve in mood disorders and eating unhealthy foods than other population groups and are less satisfied with their bodies as well as experiencing low levels of quality of life [24]. Therefore, there are significant relationships between emotional eating, mood disorders and life dissatisfaction. Due to the absence of a valid measure to assess emotional eating in Iranian society and the importance of this concept in the prevention of eating disorders as well as their treatment, it is desirable to examine the psychometric properties of the questionnaires in an Iranian sample. In order to achieve this goal, the psychometric properties of Iranian versions of the SEES and the EEQ were evaluated and compared. This study also used the Body Awareness Questionnaire (BAQ) [25] that was employed to assess divergent validity for the SEES and the EEQ in this study. Therefore, this study hypothesized that the scores of the BAQ would negatively correlate with the SEES and the EEQ.

## Method

### Participants

All participants were Iranian, lived in Iran and were fluent in Persian language. Exclusion criteria were not Iranian and not being an adult. Hair and colleagues [26] recommended a case-to-item ratio of 15:1 when conducting confirmatory factor analysis (CFA). There were 489 cases and 30 items in this study, and the minimum case-to-item ratio was met. There were 489 respondents with a mean age of 33.26 ± 9.93 who participated in this study. In terms of education, 55 (11.2%) of participants had diploma and under Diploma, 195 (39.8%) of participants had Bachelor's degree, 196 (40%) of participants had Master's degree and 43 (9%) of participants had Doctoral degree. The majority of the participants were female (90%, n = 440) and the mean Body Mass Index (BMI) of respondents was 24.41 kg/m (SD = 4.79, Range: 14.69–62.50). From the total number of participants, 28 (5.7%) were underweight (BMI < 18.5 kg/m), 276 (56.4%) were normal weight (BMI = 18.5–24.9 kg/m), 134 (27.4%) were overweight (BMI = 25.0–29.9 kg/m), and 51 (10.4%) were obese (BMI ≥ 30.0 kg/m).

### Procedure

The current goal and procedures of this study were reviewed and approved by the Alzahra University's ethics committee. The set of questionnaires were entered into Google forms and the link was sent to social networks to be completed online by the respondents. Data collection was started in June 2021 and ended in August 2021. It took participants an average of 30 min to complete the online questionnaires.

The English versions of the SEES and the EEQ were translated into the Persian language using the Brislin [27]'s approach. Both questionnaires were translated individually by two translators who were fluent in both the English and Persian languages. The questionnaires were translated from English to Persian language by one translator, who was unaware of the translation. The questionnaires were then translated back to English language by the second translator, who was oblivious to the translation. Then a comparison was made between the translated versions and the English versions and there was no contradiction between the translated versions and the English versions.

### Measures

**The Salzburg Emotional Eating Scale (SEES)** [21] is a 20 item measure that assesses emotional eating by distinguishing between various emotions and lower or higher food consumption, in reaction to the emotions. The SEES comprises four subscales with 5 items in each subscale. The four subscales are: positive (*happy subscale)*, unpleasant but low-arousal emotions (*sadness*), unpleasant but high-arousal emotions (*anger and anxiety*) and response options range from 1 (*I eat much less than usual*) to 5 (*I eat much more than usual*). Higher scores show that an individual eats more when they are stressed, whereas lower scores show that an individual eats less when they are worried. A study conducted by Meule, Reichenberger, Blechert, et al. [4] showed an acceptable internal consistency with Cronbach Alpha of 0.899.

**The Emotional Eater Questionnaire)EEQ(** [22] is a 10 item measure of emotional eating. The EEQ includes three subscales that assess (1) disinhibition, (2) type of food, and (3) guilt. Response options range from 1 (*never*) to 4 (*always*) and a lower total score indicates healthier eating behaviors. A study conducted by Garaulet and colleages [22] showed an acceptable internal consistency with Cronbach Alpha of 0.773.

**The Body Awareness Questionnaire (BAQ)** [25] comprises 18 items used to measure sensitivity in non-motor body functions (i.e., body periods and routines, the capacity to notice minor variations in normal functioning, and the ability to predict physical sensations). Response options range from 1 (*do not affect me at all)* to 9 (*they are completely true of me*), with higher total scores indicating body awareness. The original study was conducted by Shields et al. [25] and showed an acceptable internal consistency with Cronbach Alpha of 0.82. An Iranian study conducted by Taherifar et al. [28] showed an acceptable internal consistency with Cronbach Alpha of 0.88.

### Data analyses

The instruments were first evaluated for face validity, which is the degree to which end users agree that the items of an assessment instrument accurately reflect the targeted construct, as well as evaluating an item in terms of difficulty, ambiguity, and relevancy. Secondly, the content validity of the item was assessed by experts to what extent the items of instruments reflected emotional eating. Thirdly, the construct validity for each instrument was evaluated. Confirmatory Factor Analysis (CFA) using AMOS-24 software was used to assess factor loadings, measurement fit indices, convergent validity between items and internal consistency for both instruments. Before conducting the CFA, preliminary analyses such as missing data, outliers and normality were checked. The dataset had no missing values. Mahalanobis D2 was used to check outliers and the greatest Mahalanobis D2 value was not greater than 4; therefore, there was no outlier in the dataset [29]. The skewness (0.1.1 to − 0.21) and kurtosis (1.43 to 0.45) values were within the range of ± 2 and ± 3, respectively, indicating the data were normally distributed.

## Results

### Face validity

Before the data was analyzed, preliminary tests of validity were carried out on the Persian translation of the instruments. First each instrument was assessed for face validity, which is the degree to which end users agree that the items of an assessment instrument appropriately reflect the targeted construct. The face validity of the SEES and EEQ measures were qualitatively assessed during this study. Ten participants, who were not part of the current study and, were to determine the ambiguity, relevance, and difficulty of each item in the questionnaires. Examination of the respondents' opinions showed that the items in both questionnaires were understandable, relevant to the concepts, and showed no ambiguity in the vocabularies and sentences. Of the ten participants for face validity assessment, 2 of participants had Diploma, 4 of participants had Bachelor's degree, 3 of participants had Master's degree and one of participant had a Doctoral degree. Seven of the participants were females and six of them were married.

### Content validity

Ten experts (six psychologists and four counselors) were interviewed to assess the content of the SEES and EEQ. The ten experts had research and clinical experience in eating disorders. Experts were asked to comment on the essentiality, simplicity, clarity, and relevancy of each item. Experts concluded that no modifications needed to be made, based on item relevance (i.e., fit to the index aim), ambiguity (i.e., proper, and unambiguous perception of the item), and difficulty (i.e., understanding of the item).

### Construct validity

#### SEES

**Fig. 1:**
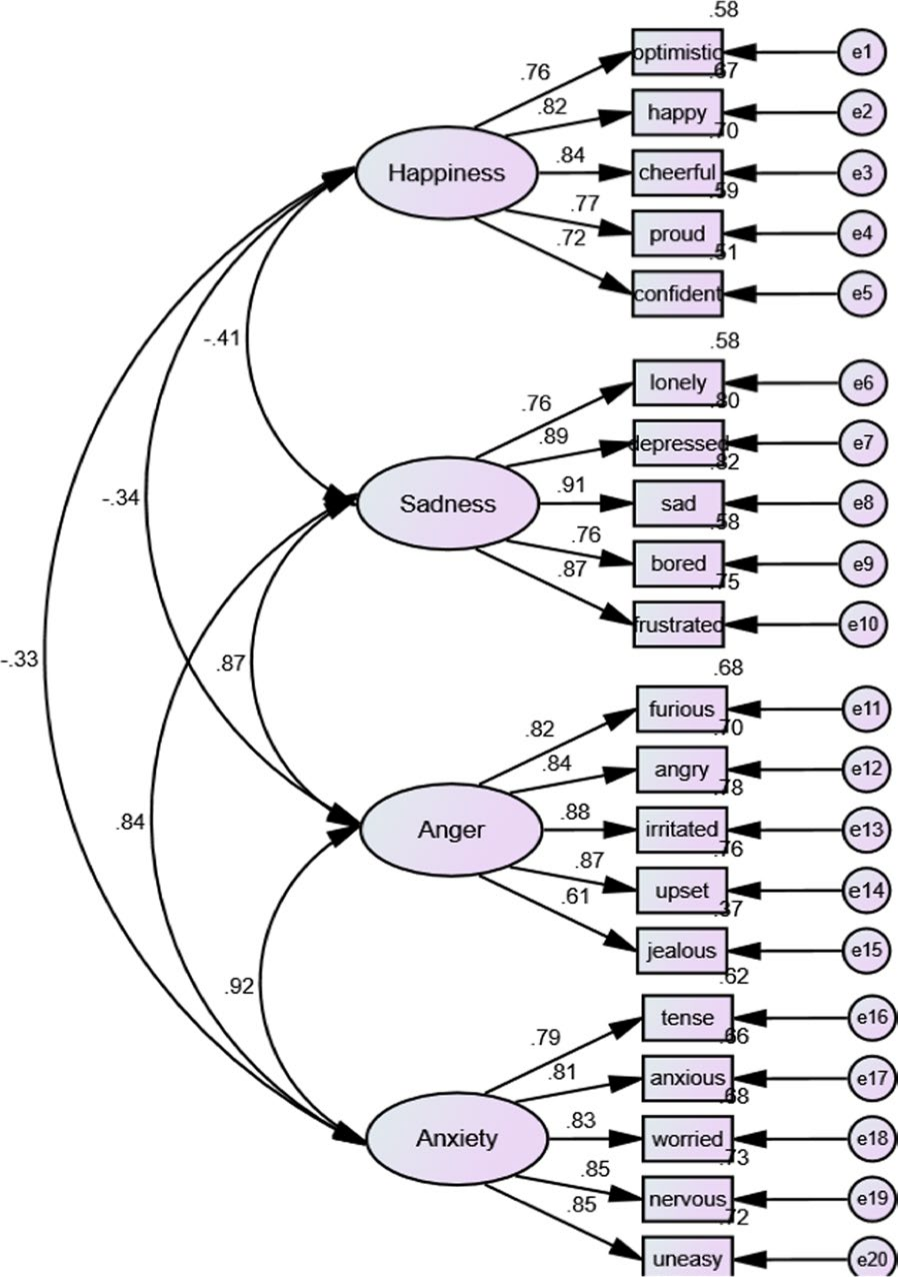
Confirmatory factor analysis of the SEES

Confirmatory factor analysis (CFA) in AMOS software (version 24) was used, and all factor loading values were greater than 0.5; therefore, all items were remained in the model (see Fig. 1).

The acceptable cut-off scores for measurement fit indices are: CMIN/DF (Chi-Square/Degree of Freedom) < 5; GFI (Goodness of Fit Index) > 0.90; CFI (Comparative Fit Index) > 0.90; TLI (Tucker-Lewis index) > 0.90 and RMSEA (Root Mean Square Error of Approximation) < 0.08 [30]. According to Byrne [30], if three of the measurement fit indices are met the cut-off scores, it can be concluded that the model has an acceptable fit. The measurement fit indices revealed the model had appropriate fit indices (CMIND/DF = 7.58, GFI = 0.91, CFI = 0.90, TLI = 0.89, RMSEA = 0.061). Average variance extracted (AVE) was used to evaluate the convergent validity and the value of AVE (0.66) was found acceptable, as it was greater than 0.5. Construct reliability (CR) was used to measure internal consistency and the value of CR (0.97) was greater than 0.9, indicating the construct had an excellent internal consistency. The Cronbach’s Alpha Coefficient value was 0.88, which was greater than the recommended cut-off score of 0.70 [31]. Table 1 shows the mean and standard deviation of each item of the SEES. The mean and standard deviation for the SEES were 2.55 and 0.94 respectively.

**Table 1:** Means and standard deviations of the items of SEES

Items	Mean	Std. Deviation
When I feel optimistic, …	2.97	0.70
When I am happy, …	3.10	0.70
When I am cheerful, …	3.15	0.64
When I am proud, …	3.02	0.60
When I feel confident, …	2.96	0.64
When I feel lonely, …	2.55	0.98
When I am depressed, …	2.38	1.11
When I am sad, …	2.29	1.04
When I am bored, …	2.39	1.00
When I am frustrated, …	2.31	0.99
When I am furious, …	2.42	1.17
When I am angry, …	2.35	1.12
When I am irritated, …	2.29	1.00
When I am upset, …	2.29	0.98
When I am jealous, …	2.72	0.79
When I am tense, …	2.57	1.01
When I am anxious, …	2.27	1.13
When I am worried, …	2.16	1.05
When I am nervous, …	2.47	1.11
When I feel uneasy, …	2.34	0.96

#### EEQ

**Fig. 2:**
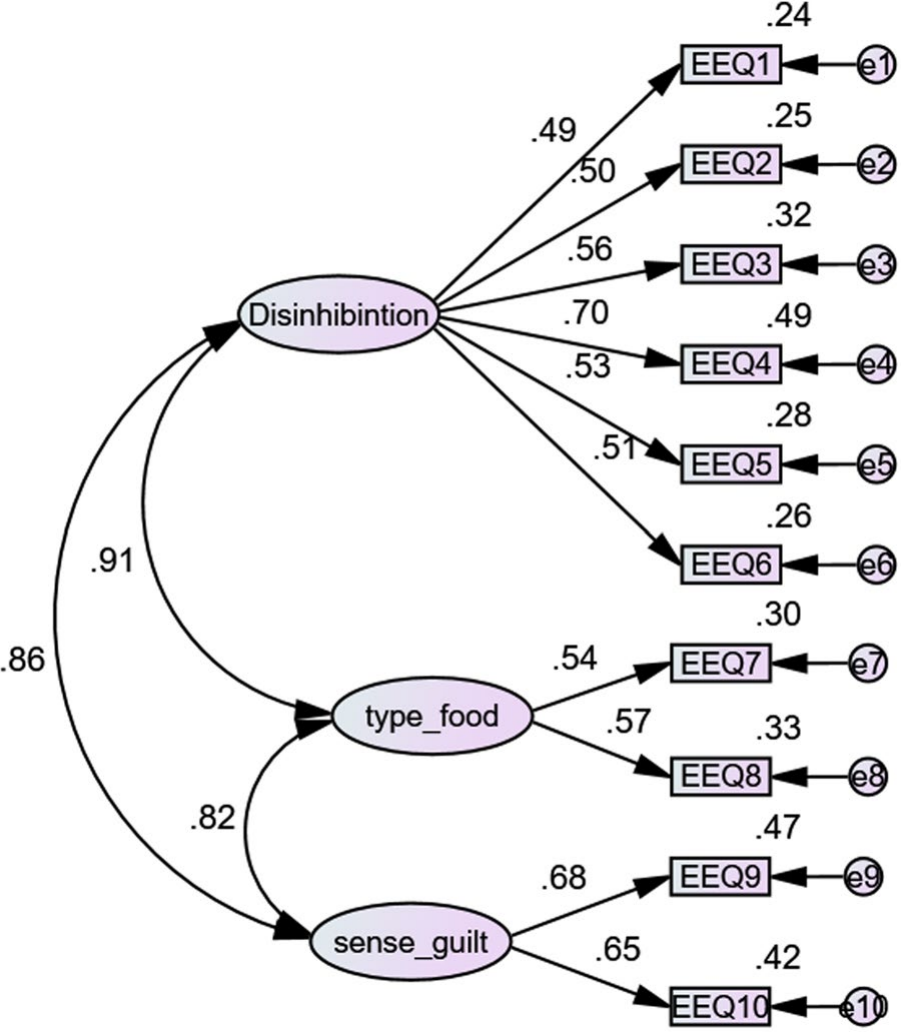
Confirmatory factor analysis of the EEQ

The result of CFA showed that all factor loading values were greater than 0.5 and, as such, all items remained in the model (see Fig. 2).

The model had appropriate fit indices (CIMND/DF = 4.15, CFI = 0.90, TIL = 0.86, RMSEA = 0.08). The AVE value of 0.33 was not acceptable, as the value was smaller than the cut-off score of 0.5. The value of CR (0.758) was greater than 0.7, indicating the construct had a good internal consistency. The Cronbach’s Alpha Coefficient value was 0.81 [31], exceeding the recommended cut-off. Table 2 shows the mean and standard deviation of each item in the EEQ. The mean and standard deviation for the EEQ were 0.95 and 0.84 respectively.

**Table 2:** Means and standard deviations of the items of EEQ

Items	Mean	SD
Do the weight scales have a great power over you? Can they change your mood?	1.20	0.89
Do you crave specific foods?	1.39	0.71
Is it difficult for you to stop eating sweet things, especially chocolate?	1.08	0.96
Do you have problems controlling the amount of certain types of food you eat?	0.87	0.83
Do you eat when you are stressed, angry or bored?	0.93	0.92
Do you eat more of your favorite food and with less control when you are alone?	0.73	0.86
Do you feel guilty when eat “forbidden” foods, like sweets or snacks?	0.88	0.92
Do you feel less control over your diet when you are tired after work at night?	0.89	0.86
When you overeat while on a diet, do you give up and start eating without control, particularly food that you think is fattening?	0.64	0.74
How often do you feel that food controls you, rather than you controlling food?	0.90	0.78

### Convergent and discriminant validity

Table 3 shows strong positive correlations between the EEQ and SEES measures in terms of convergent validity. Furthermore, correlation analysis revealed that both measures had a negative relationship with the Body Awareness Questionnaire, indicating good discriminant validity.

**Table 3:** Correlation coefficients between the studied variables (n = 489)

	1	2	3
1. EEQ	1		
2. SEES	.36**	1	
3. BAQ	− 0.04	− 0.02	1

^**^p < 0.01

## Discussion

The purpose of this study was to translate the SEES and EEQ into the Persian language and to evaluate their psychometric properties, including reliability, content and face validity, convergent validity, and discriminant validity. This study was the first to assess psychometric properties in the Persian language using an Iranian population. The Brislin method was used to translate the two measures. The results revealed that the procedure of translating both scales from the English language into the Persian language were conducted effectively, with no inconsistencies between the original versions and the translated versions. According to the findings from qualitative content validity analysis, the translated versions of both measures were appropriate for study use. The results of the face validity showed that the SEES and the EEQ have the features of transparency, relevancy, and understandability.

The results of the CFA showed that the four factors in the Persian SEES were comparable with the factors identified in the original SEES psychometric study [4]. The study results confirmed that the Persian version of the SEES consisted of four factors, which represented both positive (i.e., happiness) and negative (i.e., sadness, anger, and anxiety) emotions. The SEES, consisting of four factors, fit the data for this study, according to the goodness of fit indices. The item factor loadings were above (0.50), which is shown to be acceptable, allowing all items to remain in the SEES after translation. The CR coefficients demonstrated that the SEES had an excellent internal consistency. In addition, the AVE value of the SEES (0.66) showed that this scale had a suitable convergent validity.

The results of CFA analysis showed that the three factors in the Persian EEQ similar to the factors identified in the original EEQ psychometric study [22], comprising three factors indicative of disinhibition, type of food, and guilt. According to the goodness of fit indices, the EEQ with all three factors matched the data from the previous study well. All items remained in the scale, with item factor loadings being greater than 0.5. The CR coefficients revealed that the EEQ had a good internal consistency. Furthermore, the AVE value (0.33) suggested that this translated scale was not acceptable because the original items could not appropriately explain the latent variable used in the original study. One possible explanation for inconsistency between the current finding and the finding from the original psychometric study on EEQ [22] could be related to the participants. All participants in the original study included obese patients with a mean BMI = 31.6 ± 5.4 kg/m^2^, while in the current study only 10.4% of participants had BMI ≥ 30 kg/m^2^. Therefore, it is conceivable that low value of AVE may be due to the fact that all participants were not obese.

Regarding the comparison of the construct validity between the SEES and the EEQ, results confirmed that the SEES was better than the EEQ. This is a result of the AVE value for the SEES (0.66) being greater than the AVE value for the EEQ (0.33). Cronbach's alpha of the SEES (0.97) was greater than the EEQ (0.75), indicating better internal consistency of the SEES. The measurement fit indices of the SEES were better than the fit indices of the EEQ. Based on these analyses, the psychometric properties of the Persian SEES appear to be superior to the Persian EEQ to assess emotional eating among Iranian adults.

Consistent with our hypotheses, the results of Pearson’s correlation analysis showed that both questionnaires were positively associated with each other. Our findings exhibited negative relationships between both questionnaires using the BAQ, as such, these findings are consistent with earlier studies showing negative associations with body awareness [32, 33] and support the discriminant validity of the measures.

There are limitations in this study. First, the reliability of the two measures were calculated by CR and Cronbach's alpha; however, the test–retest method was not conducted. Future studies should evaluate the test–retest reliability for the two measures. Second, the questionnaires were completed online; as such, it is possible that some participants did not complete this study due to illiteracy or lack of access to internet. Future studies should employ the pencil and paper method to complete the questionnaires by respondents, when permitted.

## Conclusion

Notwithstanding these limitations, the current study provides initial validity and reliability evidence for two translated versions of the SEES and the EEQ to assess emotional eating in Iranian adults. The findings indicate that the SEES is better suited than the EEQ for this purpose; therefore, the SEES measure could be used by psychologists and counselors for assessing emotional eating in Persian speaking adults. The SEES could be used to evaluate and prepare early prevention programs to improve emotional eating habits.

## Data Availability

Data is available at fig share repository 10.6084/m9.figshare.16837612.
